# Drop impact onto immiscible liquid films floating on pools

**DOI:** 10.1038/s41598-024-62427-y

**Published:** 2024-06-13

**Authors:** Ben D. Fudge, Radu Cimpeanu, Alfonso A. Castrejón-Pita

**Affiliations:** 1 Department of Engineering Science, University of Oxford, Oxford, OX1 3PJ UK; 2 Mathematical Institute, University of Oxford, Oxford, OX2 6GG UK; 3 Mathematics Institute, University of Warwick, Coventry, CV4 7AL UK

**Keywords:** Fluid dynamics, Mechanical engineering, Computational science

## Abstract

The interface dynamics of a droplet impacting onto a liquid pool has been well studied, and the common interfacial velocity quantified for the cases when the pool is both the same and a different fluid to the impacting droplet. In this work we investigate, experimentally and computationally, the scenario of a droplet impacting onto a pool of the same fluid coated by a layer of another fluid with various thicknesses. The effect of the film thickness on the penetration velocity of the upper droplet-film interface is measured for experiments and simulations, and carefully compared to theoretical predictions for early-to-moderate timescales in the limiting cases of: (i) zero film thickness, in which the film has no effect and thus behaves like a fluid on same fluid impact, and (ii) infinite film thickness, in which the underlying pool has no effect. For finite layer thickness cases we carefully quantify the transition between the two limiting scenarios, and provide insight into the interfacial and flow quantities of interest, with a robust transitional behaviour observed over a rich parametric landscape. This exploration provides new quantitative insight into the nonlinear behaviour of the multi-fluid systems in newly explored finite thickness regimes, as well as a clear delineation of their effect in the context of the noted distinguished limits, with films of up to one impacting drop diameter in thickness shown to induce meaningful interpretable changes in the resulting post-impact dynamics. We also explore longer timescale features of the lower interface dynamics, revealing comparatively lower velocities and larger film thicknesses as the liquid film viscosity is increased.

## Introduction

The scenario of droplet impact onto thin films floating on deep pools is of great practical interest. Examples include raindrops impacting oil slicks on seawater can in fact spread the oil droplets much further than just the slick itself^1^ or the manufacture of encapsulated drugs^2^ in the context of advanced drug delivery systems. The introduction of a liquid film on top of an underlying deep pool adds another layer of complexity, and the possibility of further variation in parameters and resulting post-impact dynamics. Apart from the multiple liquid properties of both liquid phases, the studied multi-phase flow now includes the thickness of the film. Consequently, understanding the underlying dynamics of droplet-film-pool systems, and how varying the parameters such as the film thickness affects these dynamics, is of both fundamental and practical importance.

Previous work in this area has often focused of the dynamics of the post-impact ‘crater’ formation^3–5^, with the particular case of Zhang et al.^5^ identifying the formation of a double crown consisting of fluid from both the droplet and film for certain film thicknesses. Specifically they find an impact Weber number below which and a film thickness above which no double crown is formed, whilst in between these regimes the critical Weber number depends strongly on the film thickness, with thinner films requiring lower Weber numbers to produce a double crown. Furthermore they quantify the variation of various parameters such as both crown heights, the maximum crater volume and maximum height of the ejected jet with both the impact Weber number and film thickness providing an energetic model to explain these. Other related investigative directions include examining the formation of an air cavity behind a solid sphere impacting onto a film floating on a deep pool^6,7^ showing that an oil layer can prevent the usual sealing of the air cavity instead producing an emulsion of the oil in water, or the inverse case where an oil droplet rises in a liquid pool meeting an oil film on top and how a water layer can remain coating the droplet^8^.

One particular case of interest is the study by Kim et al.^4^, which led to finding that the maximum crater depth only depends on the film thickness for thicknesses up to $1.6\times$ the impacting droplet diameter, a critical value above which it behaves as if it were an infinitely deep pool. In contrast to the work presented here and in other examples^5,9^, therein the droplet and the film consist of the same fluid and the underlying pool of a different fluid. They further suggest that miscibility between the droplet and film could have a potential effect into the dynamics of the droplet-film-pool motion noting that in previous examples without oil layers the validity of the theoretical prediction for the crater depth depends on the spreading coefficient between the fluids and not just the immiscibility property. High-speed drop impact and the crater and crown morphology have seen renewed interest, with three-dimensional effects and experimental comparisons becoming possible in the two-fluid case, as highlighted by the extensive recent campaign by Wang et al.^10^.

Over the past five years, improved algorithmic approaches have led to considerable progress being made in three-phase flow contexts, with modelling and numerical approaches often complementing experimental insight. These range from the bursting of oil-coated bubbles as they rise towards the surface end eject droplets^11^, to impact regimes which allow the formulation of generalised predictive capabilities, from bouncing drops over thin liquid films^12^ to elucidating the motion of post-impact liquid-liquid interfaces^13^ or providing splashing thresholds in this rich parameter regime^14^. Three-dimensional drop-drop impact scenarios of different fluids have been also been recently treated computationally by Potyka and Schulte^15^, with validation work complemented by energy analysis arguments. These advances provide an excellent foundation for further development in our understanding of multi-fluid flows.

**Figure 1:**
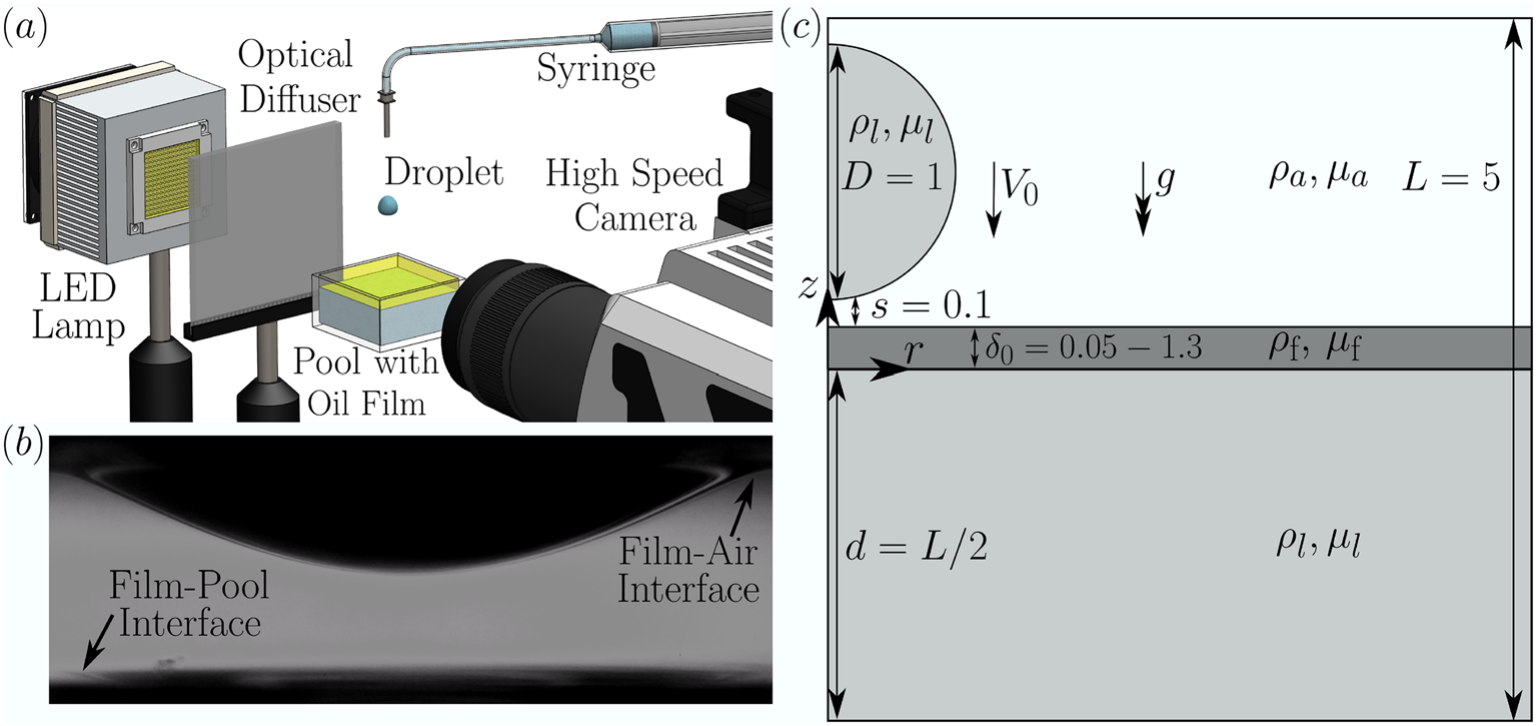
(**a**) Diagram of the experimental system with the camera aimed at the underside of the upper layer in order to capture the displacement of the drop-film interface. (**b**) Example snapshot from the impact of a $2.6\, \textrm{mm}$ diameter water droplet impacting at $0.58\, \textrm{ms}^{-1}$ onto a $350\, \textrm{cSt}$ silicone oil film showing the droplet penetrating into the film. At the bottom of the image the lower film-pool interface can be seen to be displaced slightly. (**c**) Simulation setup showing the distinction between the phases where the impacting droplet and pool are of the same fluid. The underlying pool depth is fixed at 2.5$\times$ the droplet diameter, while the thickness of the layer on top is varied whilst maintaining the distance to the droplet fixed.

Given the complex landscape described previously, the focus of this work will therefore lie in the investigation of the early time dynamics of both the upper droplet-film and lower film-pool interfaces post-impact. In particular we concentrate on how the interfacial penetration speeds vary with the film thickness for different oil film viscosities, where for the upper interface the limiting thick film case would give penetration velocities both above and below the value predicted for same fluid impact, which is approximately one half that of the impacting drop velocity^13,16–19^, whereas the immediate trend for the lower interface is less clear. Providing a quantitative basis for the observed effects of the finite thickness film on the post-impact dynamics and the thresholds beyond which it no longer exerts a significant influence on post-impact interfacial motion relative to limiting cases is a key objective of our study. We use a combination of high speed imaging experiments and high resolution direct numerical simulation (DNS) in order to find the speed of these common interfaces and carefully quantify their dependence on the film thickness for a variety of oils with different physical properties.

**Table 1:** Properties of fluids used in the simulations. $\sigma _a$ represents the surface tension of the fluid with air.

Fluid	$\rho$ $(\textrm{kg m}^{-3})$	$\mu$ $(\textrm{cP})$	$\sigma _a$ $(\textrm{mN m}^{-1})$	$\bar{V}$
Water	1000	1.0	72.0	N/A
Fluorinert FC-770	1793	1.4	14.8	0.518
$20\, \textrm{cSt}$ Silicone oil	953	19.1	20.8	0.607
$100\, \textrm{cSt}$ Silicone oil	960	96.0	20.9	0.512
$350\, \textrm{cSt}$ Silicone oil	968	338.8	21.1	0.371

The fifth column presents the theoretical normalised penetration velocity from (1) for the impact of a $1.6\, \textrm{mm}$ diameter FC-770 droplet at $0.6\, \textrm{ms}^{-1}$ onto a deep pool of the given fluid ($\text {Re}=1230$, $\text {We}=69$ and $\text {Fr}=4.8$).

## Experiment

Figure 1a illustrates the experimental setup used for this work. The impacts are captured at up to 50,000 frames per second and resolutions of up to $4\,\mu \textrm{m}$ per pixel using a single camera setup consisting of a Phantom V2512 aimed at the underside of the top of the film to capture its displacement and thus the penetration velocity. Separate images are also recorded with the droplet falling from the same height but without the presence of the pool in order to compute the droplet impact speed and diameter. In each case the impact consists of a $2.6\, \textrm{mm}$ diameter water droplet impacting at $0.58\, \textrm{ms}^{-1}$ onto a $350\, \textrm{cSt}$ silicone oil film present on top of a deep water pool. The pool and droplet consist of tri-distilled water, as this configuration provides excellent visualisation capabilities, since we are able to achieve a 90^∘^ contact angle between the interface and the perspex wall of the container when paired with the silicone oil film across a range of thicknesses from 0.05 to 1.3 times the droplet diameter, which was observed to be sufficient in order to examine the full range of variation for our quantities of interest, and to validate the numerical results. The pool container depth is greater than ten times the droplet diameter, ensuring no bottom effects are present, hence we use a constant depth of pool fluid underneath to provide a consistent baseline level. Figure 1b shows an example experiment snapshot, from which we can see the displacement of the common droplet-film interface as well as the small displacement of the lower film-pool interface at the bottom of the image. Individual frames are extracted from the videos and post processed using an in-house developed image processing toolbox written in matlab in order to find the droplet impact parameters as well as the interface penetration speed.

## Direct numerical simulation

The direct numerical simulation framework is developed using the open-source solver Basilisk^20–22^. Our setup allowed us to systematically investigate the variance in the penetration velocity across a wide range of film thicknesses. For these we use a three-phase setup^13^ in order to be able to independently vary the droplet and film properties in the presence of the surrounding air. In each case the underlying pool (which is the same fluid as the droplet) is fixed to be 2.5 times the droplet diameter to provide a constant underlying layer to eliminate any effects due to the finite depth of the pool. The film of variable depth is then initialised on top of the pool, as shown in Fig. 1c. Furthermore the domain size is sufficiently large in the lateral direction such that there are no effects such as wave reflections from the edge of the domain. In all cases the boundary conditions are solid impermeable no-slip walls at the bottom and side of the domain with a 90 degree contact angle for the side, outflow at the top of the domain and axisymmetry along the centre. Adaptive mesh refinement is used with resolutions of up to level 12 corresponding to $\sim \!820$ cells per droplet diameter with the adaptivity being based on errors in the interface location and velocity components. The simulations separately output both the top and bottom interfaces of the film in order to track their individual displacements, with the film thickness varying between 0.05 and 1.3 times the droplet diameter, in line with their experimental counterparts. For the simulations we use a pool and droplet of Fluorinert FC-770 and the films are silicone oils of either 20, 100 or $350\, \textrm{cSt}$ viscosity. These values are chosen as they provide a wide range of penetration velocities both above and below the approximately 1/2 value expected for same fluid impact, and thus we should be able to interrogate a significant variation as we change the film thickness. For the three-phase simulations we have to specify the interfacial tensions between all three pairs of fluids. For each of the liquid-air interfaces we take the surface tension to be those given in Table 1. For the interfacial tension between the silicone oils and FC-770 we use the value of $4.6\, \textrm{mNm}^{-1}$ previously measured in Fudge et al.^14^, which was found to be largely constant across a large range of silicone oil viscosities. Simulations are also performed in the case that the pool and droplet both consist of water as cross-validation to the experimental results as reported in more detail in “Experimental-numerical comparison” with the main set of results using the Fluorinert. As well as providing a large potential range of penetration velocities as reported above another advantage of using a Fluorinert droplet instead of water for the main set of results is the much lower surface tension of Fluorinert compared to water and thus larger Weber number resulting in more well conditioned as well as much less computationally expensive simulations.

We note that our observations of the impact behaviour from our experiments indicate the axisymmetric nature of the results thus justifying our use of the axisymmetric solver here. For the parameters used herein a typical run required $\sim \!15$ CPU hours on 6 CPUs with approximately $100,\!000$ grid points.

## Experimental-numerical comparison

**Figure 2:**
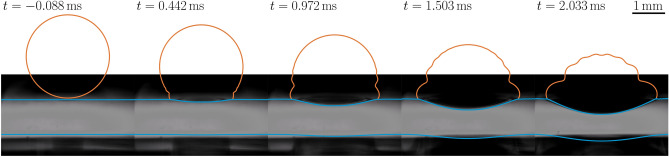
Spatiotemporal comparison between experiment and simulation for the impact of a $2.6\, \textrm{mm}$ diameter water droplet impacting at $0.58\, \textrm{ms}^{-1}$ onto a $350\, \textrm{cSt}$ silicone oil film with thickness $\delta _0/D=0.42$. The scale bar in the top right image applies throughout and the time from the theoretical point of impact if neither the droplet nor film deformed is shown for each frame. In each case the droplet profile is plotted in orange and the film in blue which overlaps with the droplet in places. The displacement of the lower film interface in the experimental images can be seen from the moving dark dimple near the bottom of the image. Supplementary videos S2–S9 for this case are available for both experiment and simulation.

In order to validate the three-phase solver for the case of impacting onto a film on a deep pool we perform a set of simulations corresponding to experiments carried out as detailed in “Experiment”. Figure 2 shows the results of one such simulation overlaid on the experimental images captured for the same parameters which in this case is a $2.6\, \textrm{mm}$ diameter water droplet impacting at $0.58\, \textrm{ms}^{-1}$ onto a $350\, \textrm{cSt}$ silicone oil film with thickness $\delta _0/D=0.42$, with the timestamps showing the time post theoretical impact if neither the droplet or pool deformed. We observe excellent qualitative agreement between the experiments and simulation data for both the deformation of the upper film droplet interface, as well as the lower film pool interface. Whilst the displacement of the lower interface cannot be directly measured at high resolution from the experimental snapshots, it is possible to see the very good agreement between numerics and experiments. The growing dark area at the bottom of the experimental images, which is also visible in Fig. 1b, act as an visual aid to see how much the bottom film has been deformed.

**Figure 3:**
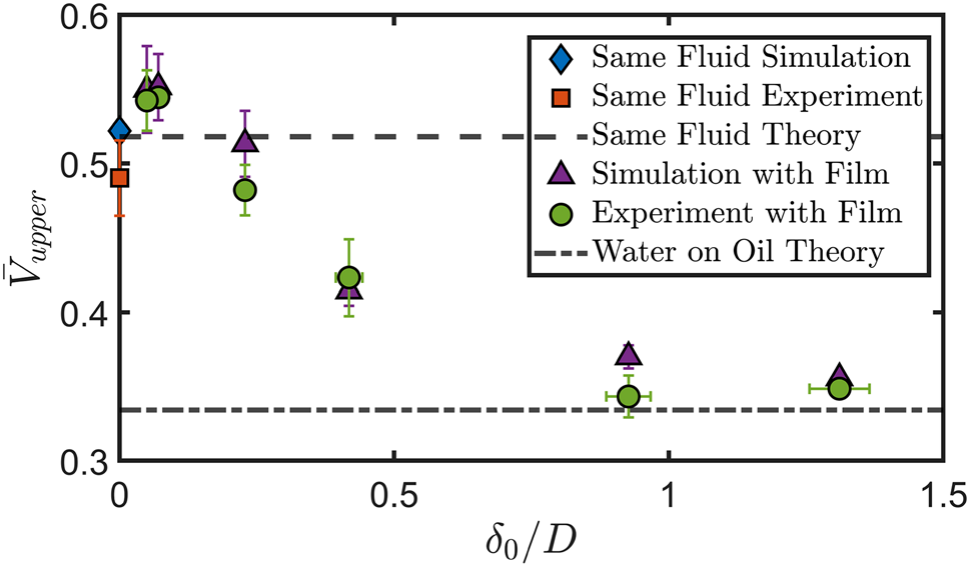
Upper interface displacement velocity $\bar{V}_{upper} = V_{upper}/V_0$ normalised by the impacting drop velocity $V_0$ in a full comparison between experiments, simulation and theory (for the limiting cases of no film thickness and infinite film thickness i.e. just water on oil given by Eq. (1), see also Fudge et al.^13^) across a wide range of film thicknesses $\delta _0$ normalised by drop diameter *D*. In each case the impact consists of a $2.6\, \textrm{mm}$ diameter water droplet impacting at $0.58\, \textrm{ms}^{-1}$ onto a $350\, \textrm{cSt}$ silicone oil film for both experiments and simulations corresponding to $\text {Re}=1470$, $\text {We}=11.8$ and $\text {Fr}=3.6$. For the experiments the error bars in the x-direction account for uncertainty in the droplet diameter as well as film thickness and the error bars in the y-direction account for the uncertainty in the pool displacement speed and droplet impact speed. For the simulations the error bars in the y-direction correspond to uncertainty in the droplet impact speed and thus the parameters used in the simulation. In some cases the error bars are smaller than the point markers.

To more comprehensively and quantitatively ascertain the agreement between the experiments and simulation we compare the experimentally measured penetration velocity $\bar{V}_{upper} = V_{upper}/V_0$, where $V_{upper}$ is the post-impact penetration velocity of the upper interface, and $V_0$ is the impacting droplet speed used as normalization factor to those predicted by their computational counterparts runs under the same parametric conditions. These results are displayed in Fig. 3, providing a summary of the penetration velocity against the normalised film thickness. The error bars in the experimental values correspond to uncertainty in the impacting droplet size (i.e. the effective frontal radius of the drop) and film thickness affecting the *x*-axis values and uncertainty in the droplet impact speed affecting the *y*-axis values. Errors in the effective radius of the droplet came from two sources (i) errors when determining the exact boundary of the droplet at the moment of impact in our image process due to the image resolution of the snapshots and imperfect background illumination and (ii) oscillations of the droplets triggered during their pinch-off from the nozzle, which varied slightly from drop to drop, introducing a small but extra experimental error on the effective radii at impact. The effect of droplet oscillations on impact and its effect on the post-impact dynamics has been investigated in detail before by Thoraval et al.^23^. Reynolds, Weber and Froude numbers, defined respectively as $\textrm{Re}=\rho _dDV_0/\mu _d$, $\textrm{We}=\rho _dDV_0^2/\sigma _a$ and $\textrm{Fr}=V_0/\sqrt{gD}$ where $\rho _d$, *D*, $V_0$, $\mu _d$ and $\sigma _a$ are the droplet density, diameter, impact speed, viscosity and surface tension of the dropet in air respectively and *g* is the acceleration due to gravity, for the corresponding simulation were set to the central value. To account for the experimental uncertainty (mostly due to the oscillations of the drop) we ran simulations at the averaged measured impact speed from the experiments, as well as $\pm 20\%$ the impact speed in order to give an estimate to the expected simulation value which are represented as the error bars in the simulation results in Fig. 3. Included is also the predicted penetration velocity in the limiting cases of infinite pool depth (for impacts onto both the same or different liquid) from the equation derived by Fudge et al.^13^, which is reproduced as expression (1) in “Discussion” below, yielding a value of 0.52 for the same fluid, and a value of 0.33 for the case of a water droplet impacting a deep pool of $350\, \textrm{cSt}$ silicone oil, both of which are shown in Fig. 3 via dashed, and dash-dotted lines, respectively. The results yet again confirm the agreement between the experiments and the simulations within the experimental error, confirming the expected trend of the penetration velocity varying between approximately 1/2 for a very thin film and that for a two-fluid setup for a very thick film. We note in both experiments and the simulations there is a slight overshoot above the 1/2 value for the smallest non-zero film thicknesses followed by a smooth decrease to an approximately constant value of $\bar{V}_{upper}\!\sim \!0.32$ for $\delta _0/D\ge 0.75$.

**Figure 4:**
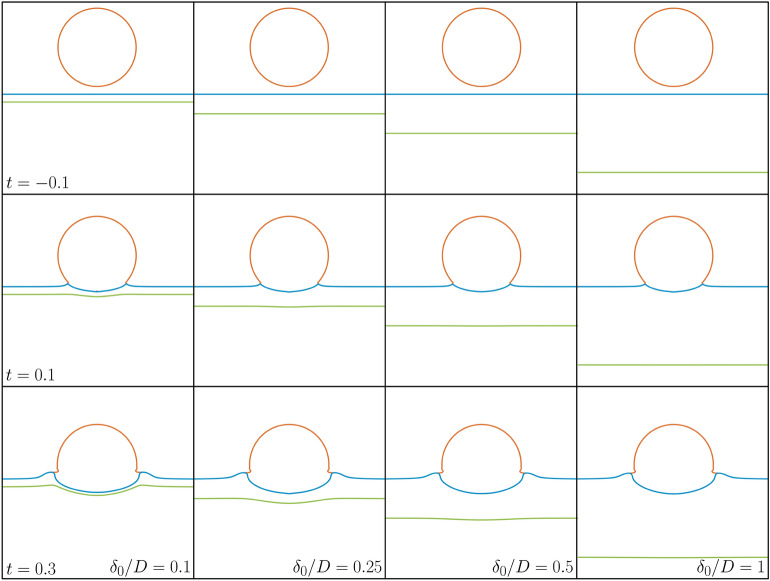
Comparison between simulations at different film thicknesses for the case of a $20\, \textrm{cSt}$ silicone oil film being impacted by a $1.6\, \textrm{mm}$ diameter FC-770 droplet at $0.6\, \textrm{ms}^{-1}$ resulting in impact parameters of $\text {Re}=1230$, $\text {We}=69$ and $\text {Fr}=4.8$. The thickness labels apply throughout each column and the time labels throughout each row with $t=0$ corresponding to the theoretical impact time if neither the droplet or film deformed. In each case the droplet interface is depicted in orange, the film upper interface in blue and film lower interface in green. Note that these images do not show the full simulation domain, with the underlying pool thickness being the same throughout but cropped for clarity. Supplementary videos S2–S9 for each of these cases are also provided.

## Discussion

Complementing our experimental campaign, we now present the results of a comprehensive sweep of simulations across several film thicknesses in the range of $0.05- 1.3$ times the droplet diameter of three different viscosity silicone oils (20, 100 or $350\, \textrm{cSt}$) for constant droplet impact conditions of $\text {Re}=1230$, $\text {We}=69$ and $\text {Fr}=4.8$, which correspond to a $1.6\, \textrm{mm}$ diameter FC-770 droplet impacting at $0.6\, \textrm{ms}^{-1}$. Table 1 presents the properties of these fluids as well as the expected penetration velocity for the impact of a FC-770 droplet onto a deep pool of each fluid at the conditions above given by Eq. (1) from Fudge et al.^13^, namely

1 $$\begin{aligned} \bar{V}_{upper}=\frac{V_{upper}}{V_0}=\frac{1}{\sqrt{1+2.71\rho _r+\frac{24.4}{\textrm{Re}}\mu _r}}. \end{aligned}$$

Here $\rho _r$ is the pool to droplet density ratio, $\textrm{Re}$ the Reynolds number based on the impacting droplet (fixed at 1230 for all cases here) and $\mu _r$ the pool to droplet viscosity ratio.

We anticipate observing differing trends for the three different viscosities. For the lowest viscosity ($20\, \textrm{cSt}$), where the penetration velocity is greater than the same fluid impact counterpart, we expect to see a decrease in the penetration velocity towards $\sim 0.5$ as the film thickness decreases whereas for the highest viscosity ($350\, \textrm{cSt}$) we expect to see an increase in penetration velocity as the film thickness decreases. For the intermediate viscosity case ($100\, \textrm{cSt}$), where the predicted penetration velocity is approximately 0.5, we expect to see a constant penetration across all film thicknesses. From a broader perspective, with all other parameters fixed, the sensitivity to the oil viscosity as predicted by equation (1) indicates the strongest variation within the selected interval, with values lower than $20\, \textrm{cSt}$ leading to negligible changes in penetration velocity, while for higher viscosity ratios between impacting drop and film of $\mathcal {O}(10^3)$ or more, the behaviour begins to saturate towards a zero value representative of the film starting to act similar to a solid non-deformable surface, albeit in a smooth gradual manner.

Figure 4 shows the time evolution of the impact onto a $20\, \textrm{cSt}$ film for four of the film thicknesses considered here, with each column representing a different thickness and time advancing down the rows with $t=0$ corresponding to the theoretical time of impact if neither the droplet nor the film deformed. In this case we expect the droplet film interface velocity to increase with increasing film thickness, which can be observed in the lowest row where the interface be be seen to have moved further down for the thickest film compared to the thinnest one. From this figure we can also see how the motion of the lower interface varies with the film thickness with noticeable deformation visible for the lowest film thickness ($\delta _0/D\!=\!0.1$) to only a slight dimple at late times for the medium thickness ($\delta _0/D\!=\!0.5$) to no visible deformation for the thickest film ($\delta _0/D\!=\!1$) visually showing the difference in the motion of the film for varying film thicknesses.

**Figure 5:**
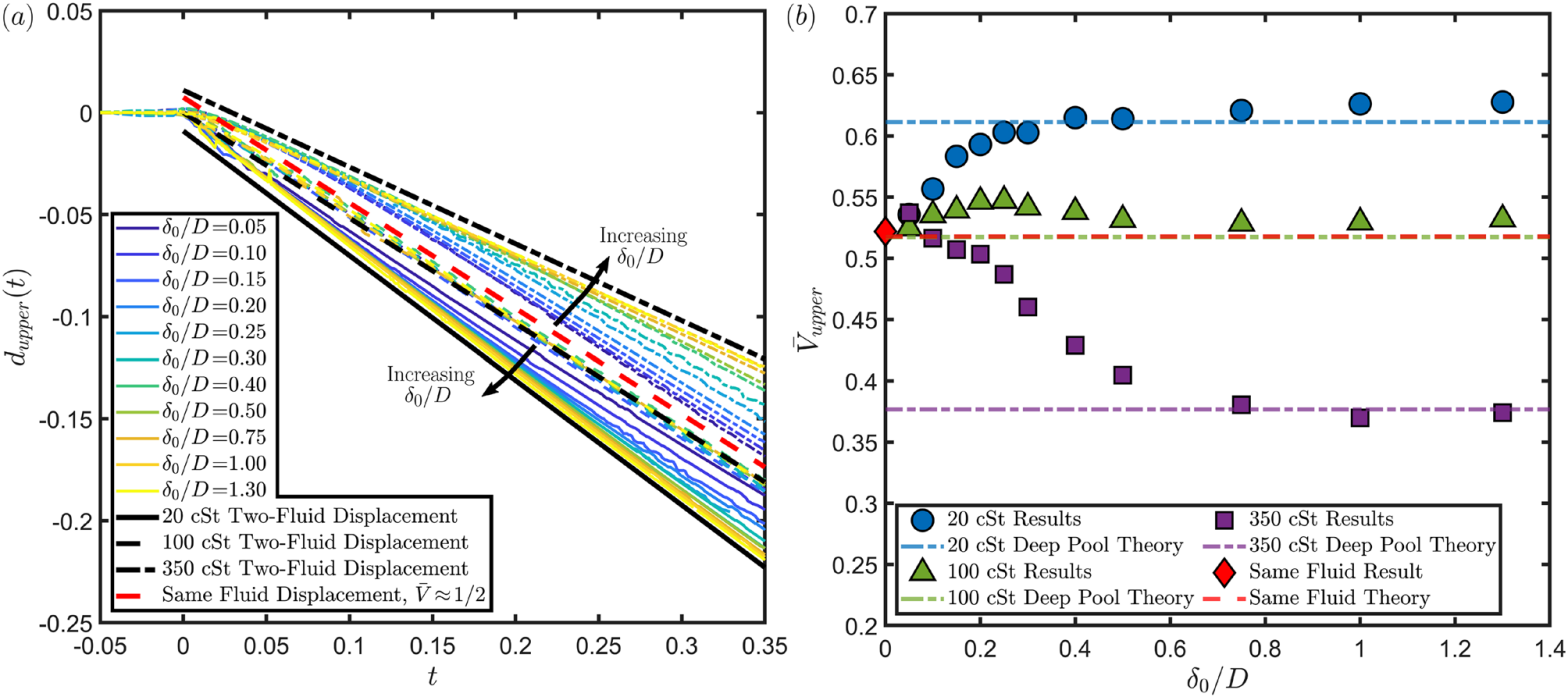
(**a**) Dimensionless displacement (normalised by drop diameter *D*) of the upper film interface against dimensionless time (normalised by $D/V_0$) for several different film thicknesses in the range $0.05\le \delta _0/D\le 1.3$ for three different film viscosities (20, 100 or $350\, \textrm{cSt}$) from simulations. In each case the impact conditions correspond to a $1.6\, \textrm{mm}$ diameter FC-770 droplet impacting at $0.6\, \textrm{ms}^{-1}$ resulting in impact parameters of $\text {Re}=1230$, $\text {We}=69$ and $\text {Fr}=4.8$. The black solid, dashed and chain lines correspond to displacement at constant speed at the theoretical penetration velocity for impact onto a pool of the corresponding silicone oils (20, 100 or $350\, \textrm{cSt}$) respectively with the lines being offset for clarity. The red line corresponds to displacement at constant speed for impact onto a pool of the same fluid, also displaced for clarity. Note that this line has the same gradient to the $100\, \textrm{cSt}$ case to within $0.1\%$. (**b**) Upper interface penetration velocity $\bar{V}_{upper}$ against the normalised film thickness $\delta _0/D$ for three different film viscosities extracted from the results in panel (**a**). Also included are dashed lines corresponding to the theoretical penetration velocities for impact onto a pool of the same silicone oil as the points of the same colour (corresponding to the black lines in panel (**a**)), calculated via the predictive formula in previous work by Fudge et al.^13^. Viscosity ratios between the liquid of the droplet and film result in penetration velocities above or below the approximately one half value pertaining to the same fluid case.

Figure 5a shows the displacement against time of the upper film surface for normalised film thicknesses in the range $0.05\le \delta _0/D\le 1.3$ for the three different film viscosities considered. From this we clearly observe the variation of the interfacial speed with the film thickness over early to moderate timescales, which we consider in reference to dynamics leading to pool deformation levels of the same order of magnitude as approximately one half of the radius of the impacting drop. Most notably for thicker films the lines are largely parallel to the corresponding lines for motion at the velocity predicted for the deep pool case (as shown by the black lines). The plot also confirms our expectation that increasing the film thickness will result in the penetration velocity tending towards the deep pool limit. We can see that for the $20\, \textrm{cSt}$ film the velocity increases with increasing $\delta _0/D$ (the solid lines) and for the $350\, \textrm{cSt}$ film the opposite is true (the chain lines). We also extract these velocities and present them in Fig. 5b confirming this trend. What we can also see from the figure is that there is a threshold thickness of the film above which we no longer distinguish any further variation of the penetration velocity with the thickness at $\delta _0/D\approx 0.5-0.75$. This suggests that this film thickness is sufficiently large to effectively act as a deep pool for the timescales considered here, and we can in fact see from Fig. 5b that above this thickness the measured penetration velocity is largely that predicted in the case of a deep pool of the oil (shown by the dashed lines). This is consistent with the results of Kim et al.^4^, where they found that the maximum crater depth formed by a droplet impacting onto a film only varies with the thickness up to thicknesses of 1.6 times the impacting droplet diameter. Whilst we have a slightly different value of the threshold here, we note that it is of the same order of magnitude and that there are differences in the scenarios such as the impact conditions, fluid arrangements (here the droplet and film are immiscible whereas in^4^ they are the same and the underlying pool different) and the timescales considered.

We also observe from Fig. 5b that for small film thicknesses in the range $0.10\!\le \!\delta _0/D\!\le \!0.3$ there is a slight upwards deviation in the penetration velocity for the $100\, \textrm{cSt}$ film case even though we expected it to be constant across all thicknesses at this viscosity. In fact a slight upwards deviation could also be considered for the 20 and $350\, \textrm{cSt}$ cases at these thicknesses too noting that if all of the points were displaced to make the $100\, \textrm{cSt}$ case constant the 20 and $350\, \textrm{cSt}$ cases would result in fairly linear variances of the penetration velocities with thickness. This consolidates our earlier observation of this deviation at low film thicknesses in the experimental results as seen in Fig. 3 where we can see that for $\delta _0/D=0.05$ and 0.07 the measured penetration velocity is larger than the theoretically predicted value^13^ of approximately 0.52 for both the experiments and simulations performed. This leads us to suggest that there is an underlying mechanism in the film motion leading to a slight increase in the penetration velocity compared to what we would expect. While this merits additional investigation beyond the scope of the current work, our initial hypothesis is linked to a spring-like mechanistic interaction while the upper and lower interfaces are in close proximity to one another. In particular, the impacting drop providing energy into the liquid film produces an initial change in both velocity and pressure inside the film which will act (on a slightly delayed timescale) on the lower interface following an early compression stage with energy build-up. As the lower interface is eventually set in motion, a recoil effect providing additional downward momentum to the entire liquid film region becomes noticeable. This is sufficient to further enhance the motion of the top interface as part of a delicate interplay between the two fluid surfaces. Access to both pressure and velocity profiles near the region of symmetry could inform a first reduced-dimensional mathematical model encapsulating the above dynamics, which we would anticipate providing qualitative (rather than quantitative) understanding of this phenomenon given some of the underlying model reduction procedures which may be required. It could nevertheless provide meaningful insight that could underpin the development of progressively more accurate data-informed variants of such a theoretical modelling framework.

**Figure 6:**
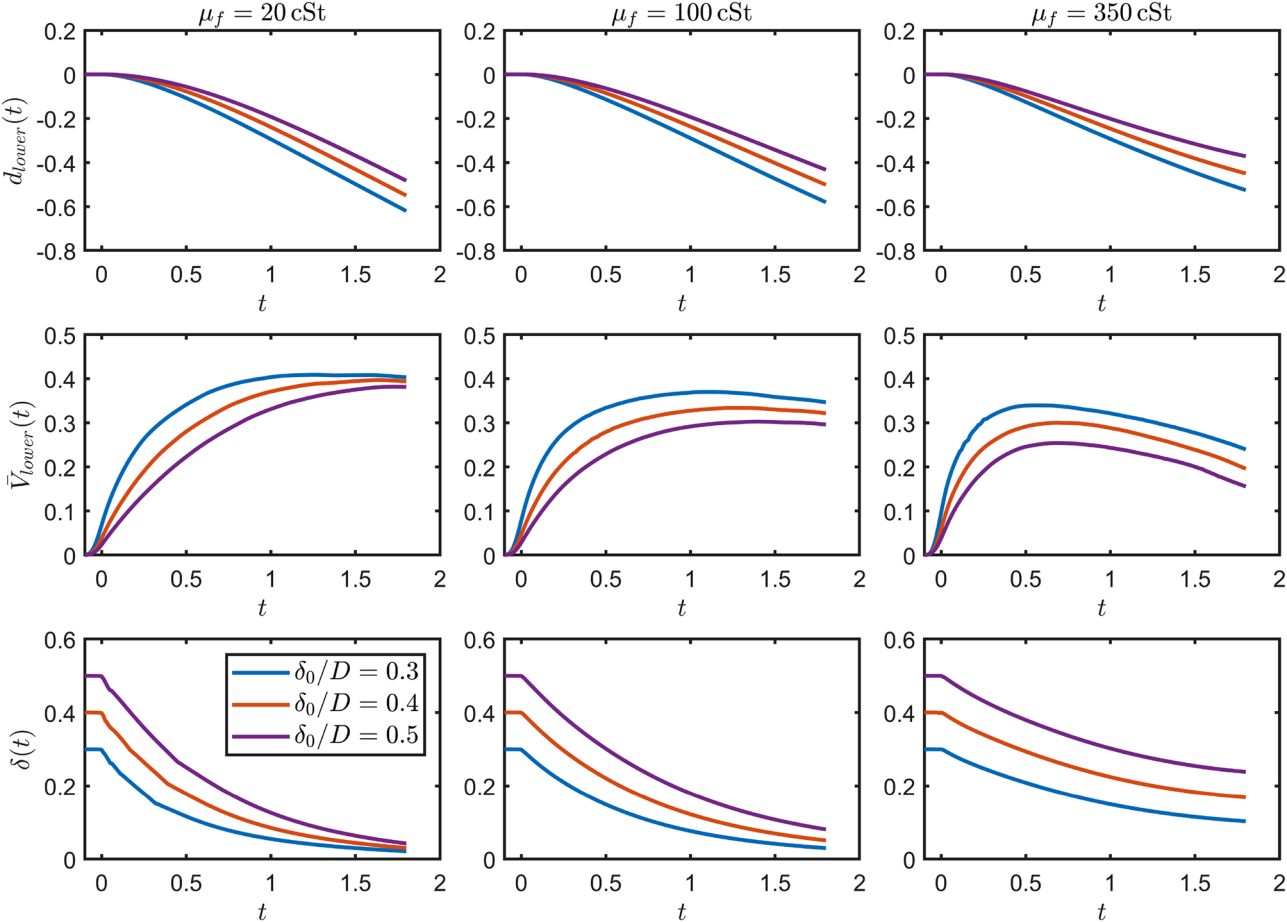
Time evolution of the displacement and velocity of the lower film-pool interface (top and middle rows respectively) and film thickness at the centre line (bottom row) for three different film viscosities (20, 100 or $350\, \textrm{cSt}$) as denoted in each column. The legend for the initial film thickness in the bottom left plot applies throughout all of the plots. In each case the impact conditions are the same as in Fig. 5, i.e. $\text {Re}=1230$, $\text {We}=69$ and $\text {Fr}=4.8$ though representing a longer simulation time with $t=0$ corresponding to the theoretical impact time if neither the droplet or film deformed. For the plot of the displacement, for each different film thickness the initial lower film-pool interface is taken to be at position $d_{lower}{=}0$ despite not being at the same location in the simulations.

Having carefully described the motion of the upper interface, we turn our attention to the lower interface dynamics, which is summarised in Fig. 6.

Early timescales proved insufficient to discern systematic behaviour in this case, and we have consequently expanded the simulation data by almost one order of magnitude in order to ascertain some of the more exquisite details. We have also restricted our attention to films of moderate thickness (up to $\delta _0/D=0.5$), as for thicker films the motion of the bottom interface (film-pool) is even more delayed and numerical experimentation has revealed negligible displacements over the relevant time scales explored here. The top row highlights the displacement of the lower film-pool interface, with the thinner films starting to move sooner, as expected due to the delay in the presence of the droplet being felt by the lower interface. As the film viscosity increases, the penetration velocity decreases as expected due to greater dissipation, with further velocity detail provided in the middle row. Interestingly, it is only for the highest viscosity pool that we can observe the penetration velocity starting to to plateau and eventually decrease within the simulation time frame considered. In the bottom row we illustrate the thickness of the oil film, i.e. the distance between the upper and lower film interfaces at the centre line. A consistent trend across all thicknesses and viscosities is revealed, with monotonic decreases towards (apparent) equilibrium values. Not unexpectedly, higher viscosities lead to an increase in both the liquid film thickness and the converge time towards it. The systematic study of the internal flow field inside the liquid film in perhaps more moderate velocity regimes reveals itself as an interesting avenue for further exploration that may help provide further predictive capabilities.

## Concluding remarks

In this work we have systematically investigated the penetration velocity of both the upper and lower interfaces of an oil film floating atop a deep pool impacted by a droplet of the same fluid as the pool. By using a range of oil film viscosities we have seen how increasing the film thickness can both increase or decrease the upper interface velocity depending on whether the deep pool limit of the oil film is greater or less than the approximately one half value for same fluids that we see when the film is very thin. The velocity tends towards the deep pool limit for increasing film thickness for all viscosities and analogous to the results of^4^ we find that there is a limit of $\delta _0/D\approx 0.5-0.75$ above which the upper penetration velocity no longer changes, with the film behaving as if it were an infinitely deep pool analogous to previous results in the literature for the depth of the crater formed during impact^4^. We have also discovered that at low film thicknesses ($0.10\le \delta _0/D\le 0.3$) we can observe a slight increase in the upper penetration velocity which we attribute to the interaction between the upper and the lower film interfaces.

Furthermore we investigated the motion of the lower film-pool interface, uncovering that for the times considered here the downward velocity decreases as the film thickness increases due to the time delay of the impact transferring through the film. This necessitated the inspection of longer timescales. We also found that the lower interface velocity only varies weakly with the oil film viscosity. A slight decrease in the velocity can be observed with increasing viscosity, which we attribute to the greater stiffness of the liquid film. The non-monotonic behaviour of the interface velocity—an early increase, followed by plateauing and an eventual decrease in velocity as the impact energy is dissipated, becomes more easily apparent in the high viscosity regime.

By combining experimental results with high resolution three-phase simulations, as well as theoretical predictions for the impact onto deep pools^13^, we have provided detailed insight into the dynamics of droplet impact onto a liquid film floating on a deep pool. Applications include mitigating oil spread after spills^1^ or the manufacture of encapsulated drugs^2^. Robust algorithmic design will further consolidate progress towards challenging regimes with even stronger viscosity contrasts or multi-scale temporal dynamics, with fluid-structure interaction scenarios such as impact onto membranes^24,25^ now also within reach. From an analytical standpoint, further computationally-assisted work will focus on the small deviation seen for small film thicknesses, with a detailed sweep of the parameters in this regime to more fully investigate this effect, or more in depth modelling of the lower interface motion to incorporate its temporal evolution and effect on the full multi-liquid system.

### Supplementary Information

Supplementary Information 1.

Supplementary Information 2.

Supplementary Information 3.

Supplementary Information 4.

Supplementary Information 5.

Supplementary Information 6.

Supplementary Information 7.

Supplementary Information 8.

Supplementary Information 9.

## Data Availability

The data from the current study is available from the corresponding author on reasonable request. The direct numerical simulation implementation supporting the findings reported in this manuscript are openly available from the GitHub repository at https://github.com/OxfordFluidsLab/Impact_on_Films.
