# Supplementary material for: Drop impact onto immiscible liquid films floating on pools
Source: Sci Rep. 2024 Jun 13;14:13671. doi: 10.1038/s41598-024-62427-y (PMC11176368; doi:10.1038/s41598-024-62427-y)
Supplement: Supplementary file 1 — Supplementary Information 1. [file 41598_2024_62427_MOESM1_ESM.pdf]

Drop impact onto immiscible liquid films floating on pools: Supplementary Information

Ben D. Fudge[1,2,*], **Radu Cimpeanu**[2,3,+], **and Alfonso A. Castrejón-Pita**[1,o]

[1]Department of Engineering Science, University of Oxford, Oxford, OX1 3PJ, UK
[2]Mathematical Institute, University of Oxford, Oxford OX2 6GG,UK
[3]Mathematics Institute, University of Warwick, Coventry CV4 7AL, UK
[*]benjamin.fudge@maths.ox.ac.uk
[+]radu.cimpeanu@warwick.ac.uk
[o]alfonso.castrejon-pita@eng.ox.ac.uk

S1 Numerical Setup and Validation

As highlighted in Section 2 of the main text, we use the three-phase numerical setup employed in Fudge et al. 2021[1] and Fudge et al. 2023[2]. The present supplementary material explores some of the key details pertinent to our present setup, while also expanding on useful verification cases and limitations of the methodology. In the particular numerical setup used herein, there is one volume-of-fluid (VOF) colour function applied to each of the three phases, each with its own assigned interfacial tension coefficient. This has the result that for each single physical interface between two phases there are in fact two VOF interfaces present, and the sum of the interfacial tension coefficients for each phase present is equal to the actual interfacial tension of the two phases. Thus for phase i out of phases i, j and k we set the interfacial tension coefficient σ_i as $(\sigma_{ij} + \sigma_{ik} - \sigma_{jk})/2$. Consequently if, for example, there was an interface between phases i and j then σ_i and σ_j would sum to σ_{ij}, the correct value.

As one verification we use the common test case of a liquid lens[3,4], wherein a spherical droplet is initialised halfway between the interface of two other fluids (commonly a liquid and air) as demonstrated in Fig. S1 (a). The droplet then deforms due to surface tension, eventually taking the shape of an elliptical lens with the angles between the three phases determined by the balance of surface tensions at the triple contact point as illustrated in Fig. S1 (b). These angles can be calculated via the Neumann triangle with the angle of each phase given by equation (1),

$$\theta_i = \cos^{-1}\left(\frac{\sigma_{\mathrm{jk}}^2 - \sigma_{\mathrm{ij}}^2 - \sigma_{\mathrm{ik}}^2}{2\sigma_{\mathrm{ij}}\sigma_{\mathrm{ik}}}\right). \tag{1}$$

The temporal dynamics of one such liquid lens setup can be seen in Fig. S1 (c) showing the time evolution of the angles between the three phases compared to those predicted by the Neumann triangle in equation (1) for the case of interfacial tensions given by σ_{dp}/σ_{da}=0.8 and σ_{pa}/σ_{da}=1.2. From the figure it can be seen that after an initial transient when the drop deforms from its spherical shape the angles converge to the predicted values and remain constant with deviations less than 0.5^{o}. This shows that the three-phase implementation is correctly capturing the balance interfacial tensions at the triple point, and its subsequent motion is reliably propagated into the implementation at the interfacial reconstruction phase, towards the expected equilibrium value. It is worth noting however that the dynamic evolution towards such a state may depend on physical considerations outside the remit of the model highlighted above, and should be given due attention should the regimes of interest fall within this framework. We also take this opportunity to highlight well-known challenges with the VOF method in scenarios in which angles are close to extreme values, with lack of accuracy anticipated should any of the contact angles reach values below approximately 15° and above 165°, and for which more specialised versions of the above algorithm should be developed. This represents an active research area in our community, with new algorithmic formulations being developed at present, or alternative methodologies (e.g. inspired by precursor films) representing viable options for particular physical setups in which it is known that such difficult regimes beyond the scope of the present work are reached.

An additional validation study conducted is that of a dynamic example, where we impact a 1.4 mm diameter Fluorinert FC-770 droplet onto a 1,000 cSt silicone oil pool at 1.72 ms^{-1}, and measure the maximum spreading distance of the droplet along the oil surface for both experiment and simulation, as illustrated in Fig. S2. Here $t = 0$ corresponds to the theoretical time of impact if neither the droplet nor pool deformed, and the gap at the earliest time is before the droplet spreading is visible. From the figure we can see excellent agreement in the spreading dynamics between experiment and simulation, with a potential cause in the slight disagreement at later times being due to uncertainties in the droplet impact speed and diameter. We are hence confident that both the delicate balancing of interfacial tensions at the triple contact point, as well as the overall three-phase dynamics are accurately captured, ensuring the accuracy and robustness of the main results discussed in this work.

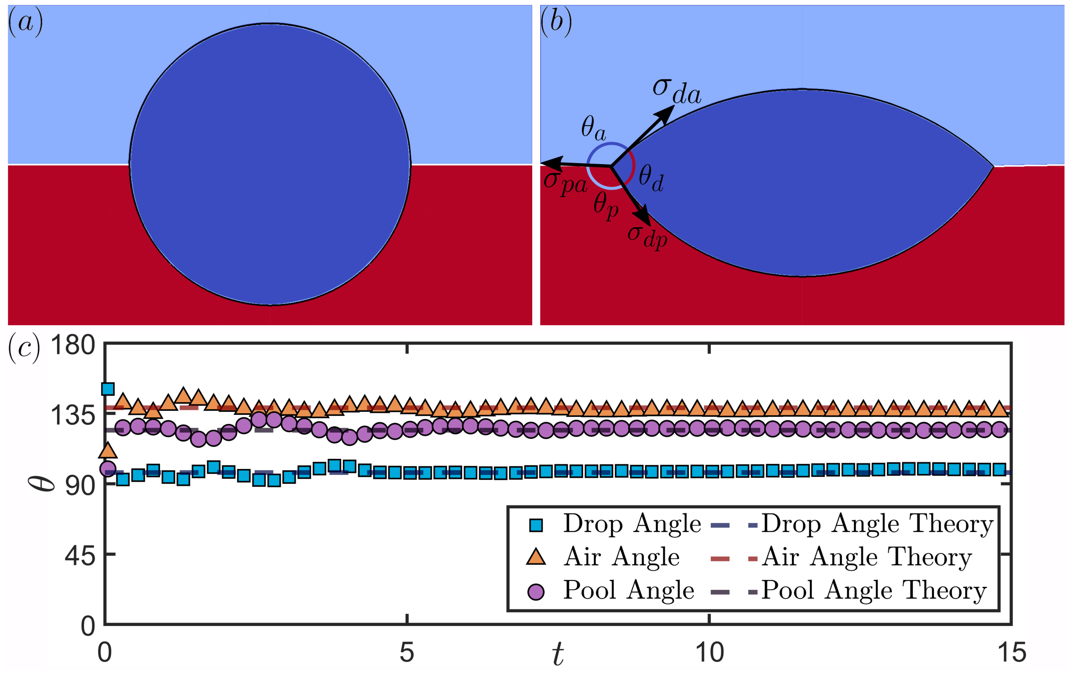

Figure S1. Liquid lens example showing the initial spherical droplet (a) and the steady state lens shape (b). For the case shown here the interfacial tensions are given by σ_{dp}/σ_{da}=0.8 and σ_{pa}/σ_{da}=1.2 denoted by the arrows where the subscripts d, p and a refer to the droplet, pool and air respectively. Note that the tensions are not drawn to scale. The subsequent theoretical droplet, pool and air angles as defined in the right-hand image are given by θ_d=99.1°, θ_p=124.4° and θ_a=136.5° respectively whereas the simulation values are θ_d=100.7°, θ_p=122.7° and θ_a=136.1° respectively all ±0.5°, a very good agreement with a less than 2% difference at most. (c) The time evolution of the angles showing an initial transient before converging to a constant value in good agreement with the theory.

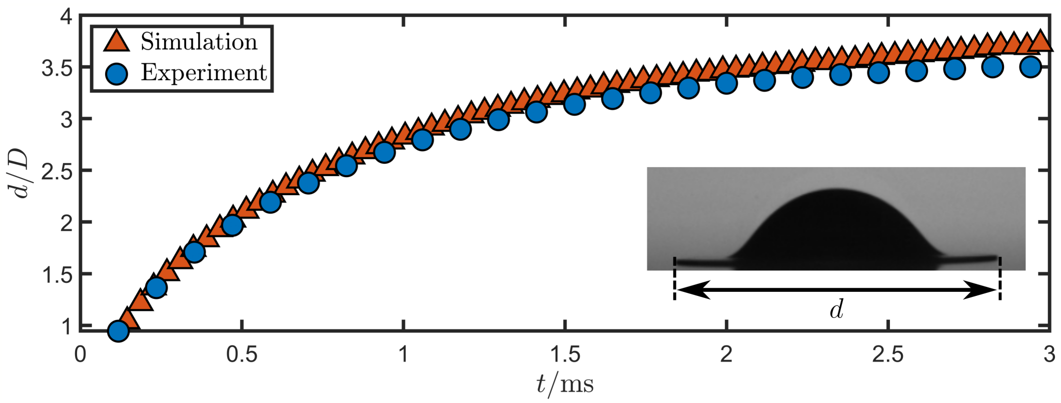

Figure S2. Comparison of the spreading distance between experiment and simulation for the impact of a 1.4 mm diameter FC-770 droplet onto a 1000 cSt silicone oil pool at $1.72\,\mathrm{ms}^{-1}$ (corresponding to Re = 3100, We = 500, Fr = 14.7) with the spreading distance as defined in the inset. Here $t = 0$ corresponds to the theoretical time of impact if neither the droplet or pool deformed. Note the dark area underneath the centre of the droplet in the inset is its reflection in the pool.

S2 Supplementary Videos

We also provide a selection of both experimental and direct numerical simulation summary videos relevant to comparisons or explorations detailed as part of Figures 2, 3 and 4 in the main document, as well as discussions associated with the respective visualisations.

References

1. Fudge, B. D., Cimpeanu, R. & Castrejón-Pita, A. A. Dipping into a new pool: The interface dynamics of drops impacting onto a different liquid. Phys. Rev. E **104**, 065102 (2021).
2. Fudge, B. D., Cimpeanu, R., Antkowiak, A., Castrejón-Pita, J. R. & Castrejón-Pita, A. A. Drop splashing after impact onto immiscible pools of different viscosities. J. Colloid Interface Sci. **641**, 585–594 (2023).
3. Yeganehdoust, F., Attarzadeh, R., Karimfazli, I. & Dolatabadi, A. A numerical analysis of air entrapment during droplet impact on an immiscible liquid film. Int. J. Multiph. Flow **124**, 103175 (2020).
4. Wang, B., Wang, C., Yu, Y. & Chen, X. Spreading and penetration of a micro-sized water droplet impacting onto oil layers. Phys. Fluids **32** (2020).
